# An 18-year, single centre, retrospective study of long-term neurological outcomes in paediatric submersion-related cardiac arrests

**DOI:** 10.1016/j.resplu.2024.100632

**Published:** 2024-04-13

**Authors:** Denne Scharink, Maayke Hunfeld, Marijn Albrecht, Karolijn Dulfer, Matthijs de Hoog, Annabel van Gils, Rogier de Jonge, Corinne Buysse

**Affiliations:** a Department of Neonatal and Paediatric Intensive Care, Division of Paediatric Intensive Care, Erasmus MC Sophia Children’s Hospital, University Medical Center Rotterdam, Doctor Molewaterplein 40, 3015 GD Rotterdam, the Netherlands; b Department of Paediatric Neurology, Erasmus MC Sophia Children’s Hospital, University Medical Center Rotterdam, Doctor Molewaterplein 40, 3015 GD Rotterdam, the Netherlands; c Department of Child and Adolescent Psychiatry/Psychology, Erasmus MC Sophia Children’s Hospital, University Medical Center Rotterdam, Doctor Molewaterplein 40, 3015 GD Rotterdam, the Netherlands

**Keywords:** Children, Resuscitation, Drowning, Long term follow-up, Outcome research

## Abstract

**Aim:**

Investigate long-term outcome in paediatric submersion-related cardiac arrests (CA).

**Methods:**

Children (age one day-17 years) were included if admitted to the Erasmus MC Sophia Children’s Hospital, after drowning with CA, between 2002 and 2019. Primary outcome was survival with favourable neurological outcome, defined as a Paediatric Cerebral Performance Category (PCPC) score of 1–3 at longest available follow-up. Secondary outcome were age-appropriate neuropsychological assessments at longest available follow-up.

**Results:**

Upon hospital admission, 99 children were included (median age at time of CA 3.2 years [IQR 2.0–5.9] and 65% males). Forty children died in-hospital (no return of circulation (45%) or withdrawal of life sustaining therapies (55%)) and 4 children deceased after hospital discharge due to complications following the drowning-incident. Among survivors, with a median follow-up of 2.3 years [IQR 0.2–5.5], 47 children had favourable neurological outcome (i.e. PCPC 1–3) and 8 children unfavourable (unfavourable outcome group total n = 52, i.e. PCPC 4–5 or deceased). Twenty-six (47%) children participated in a neuropsychological assessment (median follow-up 4.0 years [IQR 2.3–8.7]). Compared with normative test data, participants obtained worse general (*p* = 0.008) and performance (*p* = 0.003) intelligence scores, processing speed (*p* = 0.002) and visual motor integration scores (*p* = 0.0012).

**Conclusions:**

Although overall outcome in survivors was favourable at longest available follow-up, significant deficits in neuropsychological assessments were found. This study underlines the need for a standardized long term follow-up program as standard of care in paediatric drowning with CA.

## Highlights

• In all children who were admitted after an OHCA due to drowning, a high mortality rate was observed;
• Most survivors had a favorable functional outcome (PCPC) at longest available follow up;
• However, significant deficits in neuropsychological assessments were found;
• Our study emphasizes the importance of establishing a specialized follow-up program.

## Introduction

Drowning is a leading cause of paediatric out-of-hospital cardiac arrests (paediatric OHCA) and mortality in children worldwide, with an estimated global incidence of death of 7.2 per 100 000 children per year.^1^ Survivors of drowning may suffer from severe neurological and neurocognitive morbidity due to prolonged cerebral hypoxia.^2^, ^3^, ^4^, ^5^

Concluding from existing literature, it appears that deficits in neurological functioning, intelligence scores, behaviour, and health-related quality of life (HRQoL) may be observed in survivors of paediatric drowning.^2^, ^3^, ^4^, ^5^, ^6^, ^7^, ^8^, ^9^, ^10^, ^11^, ^12^ The available research is however often hampered by relative short follow-up periods or crude outcome measurements.

In 2012, the Erasmus MC Sophia Children’s Hospital implemented a standardized multidisciplinary follow-up program for all children who experienced a cardiac arrest (CA).^13^, ^14^ At this follow-up program general and neurological functioning and more detailed neuropsychological examination is performed. This program uncovered a wide range of long-term functional and neuropsychological outcomes in children who experienced an OHCA due to drowning, ranging from no deficits to severe impairments and even death. The aim of this study was to investigate survival and long-term functional and neuropsychological outcomes in this homogeneous cohort of paediatric OHCA due to drowning.

## Methods

### Study design

This single-centre retrospective cohort study was performed at the Erasmus MC Sophia Children’s Hospital, a tertiary-care University children’s hospital in the Netherlands. The hospital and Helicopter Emergency Medical Service (HEMS) provide health care in the southwest of the Netherlands with approximately five million inhabitants, about 25% of the Dutch population. Approval for this study was granted by the Erasmus MC Medical Ethics Review Board (MEC-2022–0324).

### Patient inclusion

All children, aged 1 day to 17 years, admitted to the Erasmus MC Sophia Children’s Hospital between 2002 and 2019 after OHCA due to drowning were eligible for inclusion. Children with a pre-arrest Paediatric Cerebral Performance Category (PCPC) score of greater than 3, a known neurodegenerative disease, or who suffered additional traumatic brain injury during the event were excluded.

### Outcome measures

The primary outcome measure was survival with a favourable functional outcome, defined as a Paediatric Cerebral Performance Category (PCPC) score 1–3 at the longest available follow-up period. Unfavourable outcome was defined as survival with a PCPC of 4–5 or no survival (PCPC 6). PCPC scores were assessed independently by two physician-researchers (DS, MA) and a paediatric neurologist (MH). In case of discrepancies, a consensus meeting was held to reach agreement. The secondary outcome included age-appropriate neuropsychological assessments at longest available follow-up.

### Data collection

All data were derived from emergency services registration forms and hospital records. Data included: Patient characteristics (age, gender, pre-existing illness, and socioeconomic status (SES); OHCA characteristics (basic life support (BLS), duration of resuscitation, initial rhythm and first lactate, pH and temperature after return of spontaneous circulation (ROSC)); Post-OHCA characteristics (extra corporal membrane oxygenation (ECMO), temperature management, Glasgow Coma Scale (GCS) and brain stem reflexes); Outcome (survival or cause of death (i.e. no ROSC, brain death, multi organ failure or withdrawal of life-sustaining therapies (WLST)), follow-up duration, PCPC (S1), functional status score (S2), and neuropsychological outcomes. Cardiopulmonary resuscitation (CPR) was defined as BLS, in line with the European Resuscitation Council Guidelines, and if needed, followed by advanced paediatric life support.^15^ SES was calculated using status scores from the Dutch Centraal Bureau voor de Statistiek (Statistics Netherlands) divided into tertiles.^16^ This score is based on Dutch postal codes and represents long-term household income, relative wealth, highest attained educational level, and unemployment rate per postal code area. A SES-score of 1 is considered low, 2 intermediate, and 3 high socio-economic status.

In-hospital cause of death after initial ROSC was categorized as clinical brain death or WLST due to poor neurological prognosis, refractory circulatory shock and/or respiratory failure, or recurrent CA without ROC. WLST could consist of withdrawal or no escalation of mechanical ventilation, inotropic/vasoactive support, or ECMO. No standardized WLST protocol existed during the studied period. WLST decisions were based on expert opinion on clinical assessment often in combination with brain imaging and electroencephalography.

The criteria for targeted temperature management (induced hypothermia (33–34 °C) or controlled normothermia (36–37.5 °C)) were children who remained comatose after ROSC.

Since 2012, a standardized multidisciplinary follow-up program for paediatric CA survivors was developed at our outpatient clinic as part of standard care. Children were assessed at 3–6, 12 and 24 months post-OHCA and at ages 5, 8, 12 and 17 (estimated milestone ages according to Dutch school systems). Functional outcomes were assessed during these visits by an experienced paediatric neurologist (MH) and paediatric intensivist (CB) through a semi-structured interview with children and their parents/caregivers, and physical and neurological exams. When no follow-up visit took place, these outcomes were collected from records of hospital visits with other physicians, if available. Neuropsychological outcome was assessed at 3, 6 and 24 months after OHCA by an experienced paediatric psychologist. Between 2002 and 2011, before this standardized follow-up was set up, all neuropsychological results were obtained from a cross-sectional cohort database.

Neuropsychological outcome was assessed using the following validated and age-adequate neuropsychological tests and questionnaires (see S3 for a detailed description):

1. Development and/or general intelligence (FIQ) in children: with age- appropriate versions of the Bayley Scales of Infant Development or the Wechsler Scales (BSID-III, WPPSI-IV, WISC-III, WISC-V or WAIS-IV),^17^, ^18^, ^19^
2. Performance IQ scores (PIQ), and verbal IQ scores (VIQ) with age-appropriate versions of Wechsler Scales (WPPSI-IV, WISC-III, WISC-V or WAIS-IV),^18^, ^19^, ^20^
3. Processing speed: assessed within the Wechsler Scales (WPPSI-IV, WISC-III, WISC-V or WAIS-IV),^18^, ^19^, ^20^
4. Visual motor integration: Beery Developmental Test of Visual Motor Integration (Beery-VMI),^21^
5. Parent-reported executive function: Behaviour Rating Inventory of Executive Function questionnaires (BRIEF-P or BRIEF).^22^

### Statistical analysis

Patient, OHCA, and outcome characteristics were reported using descriptive statistics. Continuous variables were reported as median with interquartile ranges. Differences between groups were tested using a Mann–Whitney U test, depending on normality of the distribution. Categorical variables were presented as number of subjects (*n*) and percentages (%). Differences were tested with the Fisher’s exact test. Individual neuropsychological test scores were converted into Z-scores by calculating the difference with the test-mean, divided by the test-SD. A negative Z-score reflects a worse score compared with the norm, except for the BRIEF questionnaire, where higher scores reflect worse functioning (i.e. more reported problems). Outcomes were compared with normative data using a one-sample *t*-test. The aim of this study was to identify association variables of functional outcome (PCPC 1–3) and neuropsychological outcomes. A two-tailed *p*-value of <0.05 was considered statistically significant. Missing data was only visualized in our results as a descriptive, no statistical analysis was applied.

## Results

### Patient sample

**Fig. 1:**
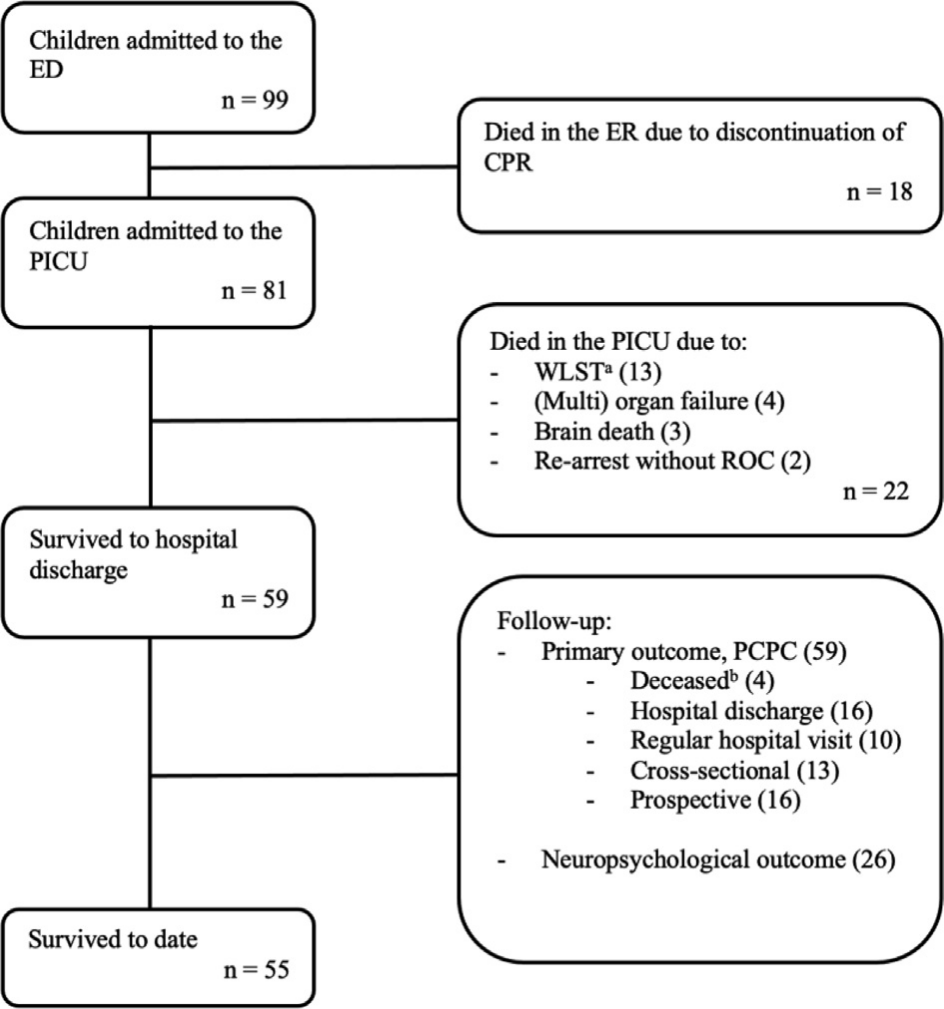
**Overview of patient inclusion.**^a^WLST specified; 8 died due to unfavorable neurological prognosis (neuro); 2 due to neuro and refractory circulatory failure (RCF) and refractory respiratory failure (RRF); 1 due to RCF and RRF; 2 not specified. ^b^Died after discharge due to complications following their drowning incident. ED = emergency department, CPR = cardiopulmonary resuscitation, PICU = pediatric intensive care unit, WLST = withdrawal of life-sustaining therapies, ROC = return of circulation.

Over the 18-year inclusion period, 99 children met the inclusion criteria. Patient and OHCA characteristics are presented in Table 1. Median age at the time of drowning was 3.2 years (IQR 2.0–5.9) and 65 (66%) were males. The overall mortality rate was 44 (44%); 18 children (18%) died in the ED due to no ROC, 22 (22%) died in the PICU mainly due to withdrawal of life-sustaining therapies (reasons for WLST (13/22) further specified in Fig. 1) and 4 children (4%) died after hospital discharge due to complications following their drowning event (Fig. 1). A detailed overview of timing and source of long-term neurological outcome is found in our supplemental files (S4).

**Table 1:** Patient and event characteristics.

	Overall	Favourable outcome (PCPC ≤ 3) (*n* = 47)	Unfavourable outcome (PCPC ≥ 5) (*n* = 52)	*p*-value	Missing *n*, (%)
Patient characteristics					
Age at event (years)	3.2 [2.0, 5.9]	2.8 [1.9, 6.1]	3.4 [2.2, 5.6]	0.470	0 (0.0)
Male gender	65 (65.7)	27 (57.4)	38 (73.1)	0.155	0 (0.0)
Pre-existing illness	20 (20.4)	12 (26.1)	8 (15.4)	0.289	1 (1.0)
SES parents	94 (94.9)	45 (95.7)	49 (94.2)	0.167	5 (5.1)
– 1 (low)	26 (27.7)	10 (22.2)	16 (32.7)	0.369	
– 2 (intermediary)	52 (55.3)	24 (53.3)	28 (57.1)	0.870	
– 3 (high)	16 (17.0)	11 (24.4)	5 (10.2)	0.119	
Event characteristics					
Events witnessed	8 (8.2)	4 (8.5)	4 (7.8)	1.000	1 (1.0)
Bystander BLS	73 (75.3)	41 (87.2)	32 (64.0)	**0.016**	2 (2.0)
CPR duration (minutes)	16.0 [4.0, 67.0]	4.0 [2.0, 8.0]	60 [27.5, 90.0]	**<0.001**	14 (14.1)
– ≤10 minutes	36 (42.4)	34 (81.0)	2 (4.7)	**<0.001**	
– 11–30 minutes	18 (21.1)	6 (14.3)	12 (27.9)	0.204	
– >30 minutes	31 (36.5)	2 (4.8)	29 (67.4)	**<0.001**	
ECPR	6 (6.1)	2 (4.3)	4 (7.7)	0.769	0 (0.0)
ROC	81 (81.8)	47 (100.0)	34 (65.4)	**<0.001**	0 (0.0)
– At scene	59 (59.6)	44 (93.6)	15 (28.8)	**<0.001**	
– At ED	22 (22.2)	3 (6.4)	19 (36.5)	**0.001**	
Post-event characteristics					
First pH after ROC*	6.9 [6.7, 7.2]	7.2 [7.0, 7.3]	6.7 [6.5, 6.9]	**<0.001**	2 (2.0)
First lactate_(mmol/L)_ after ROC*	13.4 [4.9, 16.0]	4.8 [3.0, 8.6]	15.0 [14.8, 18.8]	**<0.001**	5 (5.1)
First temperature at ED	31.7 [29.3, 34.7]	34.6 [31.1, 36.5]	30.2 [28.5, 32.6]	**<0.001**	7 (7.1)
Post-ROC ECMO	11 (11.1)	4 (8.5)	7 (13.5)	0.644	0 (0.0)
Temperature management**	43 (43.9)	17 (37.0)	26 (50.0)	0.274	1 (1.0)
Best GCS in the first 24 hours after ROC	4.0 [3.0, 15.0]	3.0 [3.0, 3.0]	15.0 [8.0, 15.0]	**<0.001**	1 (1.0)
– Eyes	1.0 [1.0, 4.0]	4.0 [3.0, 4.0]	1.0 [1.0, 1.0]	**<0.001**	
– Motoric	2.0 [1.0, 6.0]	6.0 [4.0, 6.0]	1.0 [1.0, 1.0]	**<0.001**	
– Verbal (not intubated)	5.0 [2.0, 5.0]	5.0 [5.0, 5.0]	2.0 [2.0, 2.0]	**<0.001**	
– Verbal (intubated)	68 (69.4)	18 (38.3)	50 (98.0)	**<0.001**	
Pupillary reflex present	93 (93.9)	47 (100.0)	46 (88.5)	**<0.001**	6 (6.1)
– At admission	54 (57.4)	40 (87.0)	14 (29.2)	**<0.001**	5 (5.1)
– First 24 hours	68 (73.1)	47 (100.0)	21 (45.7)	**<0.001**	6 (6.1)
– At discharge/WLST	59 (67.0)	44 (100.0)	21 (45.7)	**<0.001**	11 (11.1)
Follow-up (FU)					
Survival to hospital discharge	59 (59.6)	47 (100.0)	12 (23.1)	NA	0 (0.0)
Death after discharge	4 (4.0)	0 (0.0)	4 (7.7)	NA	0 (0.0)
FU interval (years)	2.3 [0.2, 5.5]	2.1 [0.0, 4.7]	1.9 [1.1, 6.0]	NA	0 (0.0)
Age at longest FU	6.5 [3.7, 12.2]	6.5 [3.8, 12.1]	5.0 [3.3, 8.6]	NA	0 (0.0)
PCPC at hospital discharge***	2.0 [1.0, 3.0]	1.0 [1.0, 2.0]	4.0 [4.0, 5.0]	NA	0 (0.0)
PCPC at longest FU***	2.0 [1.0, 3.0]	1.0 [1.0, 2.0]	4.0 [4.0, 5.0]	NA	0 (0.0)
FSS at longest FU***	6.0 [6.0, 8.0]	6.0 [6.0, 7.0]	14.0 [10.0, 23.0]	NA	4 (4.0)

Continuous variables were reported as median with interquartile ranges (IQR). Categorical variables were presented as number of subjects (n) and percentages (%).

PCPC = Pediatric Cerebral Performance Category, SES = socioeconomic status, BLS = basic life support, CPR = cardiopulmonary resuscitation, ECPR = extracorporeal cardiopulmonary resuscitation, ROC = return of circulation, ED = Emergency Department, ECMO = extracorporeal cardiopulmonary support, GCS = Glasgow Coma Scale, WLST = Withdrawal of Life Sustaining Therapies, FU = Follow-up, NA = not applicable, FSS = Functional Status Scale.

* Arterial and venous blood draws.

** According to hospital post-resuscitation guidelines.

*** Among survivors.

### Functional outcomes

Of the 99 patients, 47 (47%) had a favourable neurological outcome at the longest available follow-up (Table 1). Median follow-up duration among the 55 overall survivors was 2.3 years after the event (IQR 0.2–5.5) and median age at follow-up was 6.5 years (IQR 3.7–12.2). Median PCPC and Functional Status Score at longest follow-up among survivors were respectively 2.0 (IQR 1.0–3.0) and 6.0 (IQR 6.0–8.0). Median PCPC at hospital discharge and at longest follow-up were identical; 2.0 (IQR 1.0–3.0). However, changes in PCPC-score over time were observed within individual patients; 9 patients (9%) improved over time, with one child even changing from unfavourable (PCPC 4) to a favourable outcome (PCPC 3), and 9 (9%) worsened over time.

### Neuropsychological outcomes

Of the 55 overall survivors, 26 (47%) underwent neuropsychological assessment at median follow-up interval of 2.4 years post-OHCA (IQR 2.1–6.3 years) and median age of 6.3 years (IQR 4.7–12.7 years) (Table 2). A total IQ score had been calculated for all 26 children. Due to age-dependent tests, all other outcomes were not available for all patients. Twenty-nine (53%) patients did not participate because of: inability to test due to severe disability as a result of hypoxic-ischemic encephalopathy (5 (17%)), refusal (practical or emotional; 14 (48%)), age ≥ 18 years (1 (3%)), or unknown reason (9 (31%)), they were excluded from analysis. Baseline and event characteristics were not significantly different between patients who underwent the assessment and those who did not, except for age at follow-up (S5).

**Table 2:** Neuropsychological follow-up.

Neuropsychological outcomes				
	*n*	Median test score (IQR)	Median Z-score	*p*-value*
FIQ	**26**	**92 (81–101)**	**−0.53**	***0.008***
VIQ	19	100 (82–106)	0.00	*0.308*
PIQ	**19**	**89 (77–100)**	**−0.73**	***0.003***
PS	**20**	**87 (74**–**97)**	**−0.90**	***0.002***
VMI	**18**	**41 (33**–**49)**	**−0.95**	***0.012***
BRIEF	17	52 (45–63)	0.10	*0.363*

FIQ = full scale IQ; VIQ = verbal IQ; PIQ = performance IQ; PS = processing speed; VMI = visual motor integration.

* *p*-value in comparison with normative data.

The assessed patients obtained worse scores in full scale IQ (FIQ; z = −0.53, *p* = 0.008), performance IQ (PIQ; z = −0.73, *p* = 0.003), processing speed (PS; z = −0.90, *p* = 0.002), and visual motor integration (VMI; z = −0.95, *p* = 0.012) when compared with normative data (Table 2). Of these children, 9 (35%) obtained full-scale IQ scores of 85 or below which is ≥ 1 SD below population mean.

## Discussion

Over an 18-year period, this single-centre study investigated paediatric OHCA due to drowning and found that 47% of the patients survived with a favourable functional outcome (i.e. PCPC 1–3) after a median 2.3 years (IQR 0.2–5.5) of follow-up. Further tested drowning survivors scored worse on full-scale IQ, performance IQ, processing speed, and visual-motor integration scores compared to normative data.

### Functional outcomes

Children who experience OHCA due to drowning have high mortality rates upon hospital arrival, with or without ROC (overall mortality 44%). However, survivors had an overall favourable outcome (i.e. PCPC 1–3) at longest available follow-up. While previous studies on outcomes after drowning differ in patient inclusion, follow-up interval, and sample size, they roughly show similar results.^2^, ^3^, ^4^, ^5^, ^6^, ^7^, ^8^, ^9^, ^12^

In our cohort, individual PCPC scores at hospital discharge varied over years of follow-up. This is in contrast with existing literature where it is suggested mean PCPC scores may be used to predict long-term functional outcome.^23^, ^24^, ^25^ Important to note is that in the 18 children who had a change in PCPC score at follow-up, there was 1 child who changed from an unfavourable outcome at hospital discharge (PCPC 4; dependent on others for daily support) to a favourable outcome at follow-up (PCPC 3; sufficient cerebral function for age-appropriate independent activities). These changes in outcome stress the importance of a specialised follow-up program so certain changes may be detected in time and individual treatment plans can be made.

The PCPC-score is a crude measurement tool, focused on daily activity of school-going children. Its use in research grew to be custom likely due to the easy application and interpretation. However, many paediatric drowning victims are too young to accurately differentiate between adjacent scores, since most do not yet attend school and are still dependent in performing daily activities. Furthermore, improvement in PCPC could be seen due to the plasticity of a child’s brain, which allows recovery even years after cerebral hypoxia.^26^, ^27^, ^28^ Conversely, a decrease in PCPC score could be explained by the “growing into deficit” phenomenon. Children with acquired brain injury may not show deficits until later in life when more cognitive demands are placed on them.^26^, ^27^, ^28^ Our results further emphasize the importance of standardized long-term follow-up into adulthood, in large homogeneous patient samples to obtain an adequate assessment of outcome, especially participation in society, cognitive functioning and quality of life.^29^

Kieboom et al. investigated the correlation between CPR-duration and neurological outcomes one year after the event in hypothermic (core body temperature < 34 °C) drowning patients using the PCPC-score.^2^ Their findings suggested a higher likelihood of favourable outcomes (PCPC 1–3) when spontaneous circulation resumed within 30 minutes of Advanced Paediatric Life Support (APLS). In their cohort all hypothermic children who were resuscitated > 30 minutes had unfavourable outcome at one year follow-up. This raises doubt about the therapeutic value of resuscitation > 30 minutes in hypothermic paediatric drowning patients. However, in our cohort two hypothermic patients survived with favourable outcome after extended periods of resuscitation (40 and 90 minutes). This highlights the challenge of accurate decision-making at scene or ED, where the most important element of the discussion is the number needed to treat; how many children do you have to resuscitate for longer than 30 minutes to get one with a good outcome, compared to the number of children who might survive but with a very poor neurological outcome and quality of life.

### Neuropsychological outcomes

Children who survived an OHCA caused due to drowning obtained worse intelligence scores compared to normative test data. This correlates with previous literature, although other studies have used a range of neuropsychological assessment methods.^6^, ^7^, ^8^, ^9^ Furthermore, 33% of the children in our cohort obtained full scale IQ scores of 85 or below (−1 SD). In the general population, this percentage is expected to be 16%. Remarkably, the children in our cohort displayed a worse performance IQ, whereas their verbal IQ score was similar to the norm. They also exhibited slower processing speed and a worse score on visual-motor integration, indicating cognitive weaknesses that may impact their school performance and overall development. However, parents did not report more executive functioning problems in the daily life of these children, based on the parent-reported BRIEF scores. The assessment of each test result must consider the broader context of other test outcomes as well as potential deficits in other domains.^7^, ^30^ These findings further highlight the need for specialized and standardized follow-up programs, ideally on a large scale.

In 2021, Hunfeld et al. published a study examining the association between PCPC scores and neuropsychological outcomes at 3–6 and 24 months after a paediatric OHCA.^13^ Consistent with our results, they found that although general functional outcomes may be favourable at follow-up, deficits in neuropsychological functioning are still present. The study concluded that PCPC scores were not associated with intelligence scores and that individualized follow-up should include neurological and neuropsychological assessments, as well as care from an educational psychologist to monitor these children’s development into adulthood and provide parents with a realistic view of their child’s intellectual strengths and weaknesses.

## Future directions

Predicting outcomes in this patient population is a challenge, as no international neuro-prognostication guideline exists due to the limited availability of data.^5^, ^12^, ^29^, ^31^, ^32^ Having precise individualized outcome predictions would aid in clinical decision-making regarding treatment options. Therefore, it is crucial to conduct further research to clarify the neurological and neuropsychological consequences. Unfortunately, our sample size was too small to develop a clinical prediction model. Multicentre (international) collaborations are needed to increase cohort size.

## Strengths and limitations

Among the strengths of our study is our representative study population and the low rate of missing variables (less than 10% per variable). Additionally, our cohort was homogeneous including solely drowning paediatric OHCA patients. Most importantly, our research consists of one of the longest and most extensive follow-ups there is on the subject.

However, our study also has several limitations: first, due to the retrospective design, and our goal to collect as much relevant data as possible, it was inevitable to use different data sets. Second, a selection bias was possibly created due to not including children who died at the scene or who were transferred to other hospitals. Third, no detailed pre-hospital resuscitation data and no pre-arrest neuropsychological assessments were available. However, it is important to note that “before drowning IQ” of very few children is probably known and thus difficult to exclude that low IQ scores were already existing before the drowning happened. Lastly, larger cohorts are needed to make prediction and prognostication possible.

## Conclusions

Although high mortality rates were still observed (44%), 85% of survivors of drowning with OHCA had favourable functional outcomes (PCPC 1–3) at longest available follow-up. Nonetheless, significant deficits in neuropsychological assessments were found. Our study emphasizes the importance of a specialized follow-up program for paediatric drowning patients throughout their young adulthood, as standard of care.

## Funding

No funding was received for this research.

## CRediT authorship contribution statement

**Denne Scharink:** Writing – review & editing, Writing – original draft, Visualization, Methodology, Investigation, Formal analysis, Conceptualization. **Maayke Hunfeld:** Writing – review & editing, Supervision, Resources, Methodology, Investigation, Conceptualization. **Marijn Albrecht:** Writing – review & editing, Resources, Investigation, Conceptualization. **Karolijn Dulfer:** Writing – review & editing, Validation, Methodology, Formal analysis, Conceptualization. **Matthijs de Hoog:** Writing – review & editing, Conceptualization. **Annabel van Gils:** Writing – review & editing, Conceptualization. **Rogier de Jonge:** Writing – review & editing, Validation, Methodology, Formal analysis, Conceptualization. **Corinne Buysse:** Writing – review & editing, Visualization, Supervision, Resources, Methodology, Investigation, Conceptualization.

## Declaration of competing interest

The authors declare that they have no known competing financial interests or personal relationships that could have appeared to influence the work reported in this paper.

## References

[1] World Health Organization. Global Report on Drowning: Preventing a leading killer. 2014.

[2] Kieboom J.K., Verkade H.J., Burgerhof J.G. (2015). Outcome after resuscitation beyond 30 minutes in drowned children with cardiac arrest and hypothermia: Dutch nationwide retrospective cohort study. BMJ.

[3] Raess L., Darms A., Meyer-Heim A. (2020). Drowning in children: retrospective analysis of incident characteristics, predicting parameters, and long-term outcome. Children (Basel).

[4] Shenoi R.P., Koerner C.E., Cruz A.T. (2016). Factors associated with poor outcome in childhood swimming pool submersions. Pediatr Emerg Care.

[5] Suominen P.K., Vahatalo R. (2012). Neurologic long term outcome after drowning in children. Scand J Trauma Resusc Emerg Med.

[6] Suominen P.K., Vahatalo R., Sintonen H., Haverinen A., Roine R.P. (2011). Health-related quality of life after a drowning incident as a child. Resuscitation.

[7] Suominen P.K., Sutinen N., Valle S., Olkkola K.T., Lonnqvist T. (2014). Neurocognitive long term follow-up study on drowned children. Resuscitation.

[8] Slomine B.S., Nadkarni V.M., Christensen J.R. (2017). Pediatric cardiac arrest due to drowning and other respiratory etiologies: Neurobehavioral outcomes in initially comatose children. Resuscitation.

[9] Manglick M.P., Ross F.I., Waugh M.C., Holland A.J.A., Cass D.T., Soundappan S.S.V. (2018). Neurocognitive outcomes in children following immersion: a long-term study. Arch Dis Child.

[10] Salas Ballestin A., de Carlos Vicente J.C., Frontera Juan G. (2021). Prognostic factors of children admitted to a pediatric intensive care unit after an episode of drowning. Pediatr Emerg Care.

[11] Al-Qurashi F.O., Yousef A.A., Aljoudi A. (2019). A review of nonfatal drowning in the pediatric-age group: a 10-year experience at a university hospital in Saudi Arabia. Pediatr Emerg Care.

[12] Quan L., Bierens J.J., Lis R., Rowhani-Rahbar A., Morley P., Perkins G.D. (2016). Predicting outcome of drowning at the scene: A systematic review and meta-analyses. Resuscitation.

[13] Hunfeld M., Dulfer K., Rietman A. (2021). Longitudinal two years evaluation of neuropsychological outcome in children after out of hospital cardiac arrest. Resuscitation.

[14] Albrecht M., de Jonge R.C.J., Nadkarni V.M. (2021). Association between shockable rhythms and long-term outcome after pediatric out-of-hospital cardiac arrest in Rotterdam, the Netherlands: An 18-year observational study. Resuscitation.

[15] Topjian A.A., Raymond T.T., Atkins D. (2020). Part 4: Pediatric basic and advanced life support: 2020 American Heart Association guidelines for cardiopulmonary resuscitation and emergency cardiovascular care. Circulation.

[16] Statistics Netherlands 2020 [Available from: https://www.cbs.nl/en-gb].

[17] Smrkovsky M. (2002).

[18] Hendriksen J.H.P. (2009).

[19] Wechsler D., Kort W., Schittekatte M., Dekker P.H., Verhaeghe P., Compaan E.L. (2005).

[20] Wechsler D. (2012).

[21] Beery K.E., Beery N.A. (2004).

[22] Smidts D., Huizinga M. (2009).

[23] Goto Y., Maeda T., Nakatsu-Goto Y. (2014). Decision tree model for predicting long-term outcomes in children with out-of-hospital cardiac arrest: a nationwide, population-based observational study. Crit Care.

[24] Michiels E.A., Dumas F., Quan L., Selby L., Copass M., Rea T. (2013). Long-term outcomes following pediatric out-of-hospital cardiac arrest*. Pediatr Crit Care Med.

[25] Silverstein F.S., Slomine B.S., Christensen J. (2016). Functional outcome trajectories after out-of-hospital pediatric cardiac arrest. Crit Care Med.

[26] Middleton J.A. (2001). Practitioner review: psychological sequelae of head injury in children and adolescents. J Child Psychol Psychiatry.

[27] van Zellem L., Buysse C., Madderom M. (2015). Long-term neuropsychological outcomes in children and adolescents after cardiac arrest. Intensive Care Med.

[28] Aarsen F.K., Paquier P.F., Reddingius R.E. (2006). Functional outcome after low-grade astrocytoma treatment in childhood. Cancer.

[29] Topjian A.A., Scholefield B.R., Pinto N.P. (2020). P-COSCA (Pediatric Core Outcome Set for Cardiac Arrest) in children: an advisory statement from the International Liaison Committee on Resuscitation. Circulation.

[30] Vanagt W.Y., Wassenberg R., Bierens J.J. (2014). No gold standard for neurocognitive outcome assessment of drowned children. Resuscitation.

[31] Hunfeld M., Muusers M.A.C., Catsman C.E., Castillo J.D., Tibboel D., Buysse C.M.P. (2020). The current practice regarding neuro-prognostication for comatose children after cardiac arrest differs between and within European PICUs: A survey. Eur J Paediatr Neurol.

[32] Geocadin R.G., Callaway C.W., Fink E.L. (2019). Standards for studies of neurological prognostication in comatose survivors of cardiac arrest: a scientific statement from the American Heart Association. Circulation.

