# Supplementary material for: An 18-year, single centre, retrospective study of long-term neurological outcomes in paediatric submersion-related cardiac arrests
Source: Resusc Plus. 2024 Apr 13;18:100632. doi: 10.1016/j.resplu.2024.100632 (PMC11026833; doi:10.1016/j.resplu.2024.100632)
Supplement: Supplementary data 1 [file mmc1.docx]

Supplementary files

**S1. Pediatric Cerebral Performance Category score (PCPC)**

| Score | Category | Description |
| --- | --- | --- |
| 1 | Normal | Normal; at age-appropriate level; |
| 2 | Mild disability | Conscious, alert, and able to interact at age-appropriate level; school-age child attending regular school classroom, but grade perhaps not appropriate for age; possibility of mild neurologic deficit |
| 3 | Moderate disability | Conscious; sufficient cerebral function for age-appropriate independent activities of daily life; school-age child attending special education classroom and/or learning deficit present. |
| 4 | Severe disability | Conscious; dependent on others for daily support because of impaired brain function |
| 5 | Coma or vegetative state | Any degree of coma without the presence of all brain death criteria; unaware, even if awake in appearance, without interaction with environment; cerebral unresponsiveness and no evidence of cortex function (not aroused by verbal stimuli); possibility of some reflexive response, spontaneous eye-opening, and sleep-wake cycles |
| 6 | Brain death | Apnea, areflexia, and/or electroencephalographic silence |

**S2. Functional Status Scale (FSS)**

|  | 1 (Normal) | 2 (Mild dysfunction) | 3 (Moderate dysfunction) | 4 (Severe dysfunction) | 5 (Very severe dysfunction) |
| --- | --- | --- | --- | --- | --- |
| Mental status | Normal sleep/wake; appropriate responsivity | Sleepy but arousable  to  noise/touch/movement and/or periods of social nonresponsivity | lethargic and/or irritable | Minimal  arousal to stimulus (stupor) | Unresponsive and/or  Coma and/or Vegetative |
| Sensory | Intact hearing and vision and responsive to touch | Suspected hearing or Suspected vision loss. | Not reactive to auditory stimuli or Not reactive to visional stimuli | Not reactive to auditory stimuli and Not reactive to visional stimuli | Abnormal response to pain or touch |
| Communication | Appropriate non-crying vocalizations, interactive facial expressiveness, or gestures | Diminished  Vocalization  Diminished Facial Expression and/or social responsiveness | Absence of attention getting behavior | No demonstration of discomfort | Absence of communication |
| Motor function | Coordinated body movements and Normal muscle control and Awareness of action and why it’s being done | 1 limb functionally impaired | 2 or more limbs functionally impaired | Poor head control | Diffuse Spasticity,  Paralysis,  Decerebrate/Decorticate  Posturing |
| Feeding | All food taken by mouth with ageappropriate help | NPO or need for ageinappropriate help with feeding | Oral and tube feedings | Parenteral Nutrition with oral or tube feedings | All parenteral nutrition |
| Respiratory | Room air and no artificial support or aids | Oxygen and/or Suctioning | Tracheostomy | CPAP for all or part of the day and/or Mechanical ventilator support for  part of the day | Mechanical ventilatory support for all of the day and night |

**S3. Overview of neuropsychological tests**

| Measurements | Test | Age  (yrs.) | Test mean, standard deviation | Notes |
| --- | --- | --- | --- | --- |
| Intellectual functioning | Bayley Scales of Infant  Development (BSID-2 or Bayley-3) | 0-2.5 | 100, 15 (1) | Intelligence or developmental quotient standard scores. Higher scores represent better functioning. Due to the small sample size, the outcomes of the different tests are grouped: Total IQ score is based on  BSID-cognitive score, WPPSI-  III TIQ, WISC-III TIQ, or WAIS-IV TIQ. The Verbal IQ score is based on: WPPSI-III  VIQ, WISC-III VIQ, WAISIV VC-index. The  performance IQ score is based on: WPPSI-III PIQ, WISC-III  PIQ, WAIS-IV PO-index |
|  | Wechsler Preschool and Primary Scale of Intelligence (WPPSI-IV) | 2.6-6 | 100, 15 (2) |  |
|  | Wechsler Intelligence Scale for Children (WISC-III-IV) | 7-15 | 100, 15 (3) |  |
|  | Wechsler Adult Intelligence Scale (WAIS-IV) | 16-18 | 100, 15 (4) |  |
| Processing speed | Age-appropriate versions of the  Wechsler Scales (WPPSI-IV,  WISC-III-IV, WAIS-IV) | ≥4 | 100, 15 (2-4) | Standard norm score: Higher scores represent better functioning |
| Visual-Motor Integration | Beery Developmental Test of  Visual Motor Integration (BeeryVMI) | ≥2 | 100,  15 (5) | Standard norm score: Higher scores represent better functioning |
| Parentreported executive function questionnaire | Behaviour Rating Inventory of  Executive Function questionnaires  (BRIEF-P and BRIEF) | ≥2 | 50, 10 (6) | Z-score; A higher score means worse functioning (more reported problems) |

**S4. Timing and source of long-term neurological outcome**

|  | **Deceased after discharge** | | **Scored at hospital discharge** | | **Scored at regular hospital or clinic visit** | | **Scored at cross-sectional follow-up (2002-2013)** | | **Scored at prospective follow-up (2011-2019)** | |
| --- | --- | --- | --- | --- | --- | --- | --- | --- | --- | --- |
| **Age at follow-up** | 4.8 [3.3, 6.8] | | 4.9 [2.0, 8.4] | | 4.0 [2.9, 6.2] | | 12.7 [8.5, 13.4] | | 8.2 [5.6, 10.8] | |
| **Follow-up years** | 1.2 [0.8, 2.7] | | 0.0 [0.0, 0.0] | | 2.0 [0.9, 4.4] | | 9.1 [3.7, 10.5] | | 2.7 [2.2, 5.6] | |
| **PCPC score** | **Pre-arrest** | **Follow-up** | **Pre-arrest** | **Follow-up** | **Pre-arrest** | **Follow-up** | **Pre-arrest** | **Follow-up** | **Pre-arrest** | **Follow-up** |
| **1 – Normal** | 4 | 0 | 13 | 10 | 9 | 5 | 11 | 3 | 15 | 4 |
| **2 – Mild disability** | 0 | 0 | 1 | 4 | 0 | 1 | 1 | 4 | 1 | 8 |
| **3 – Moderate disability** | 0 | 0 | 2 | 2 | 1 | 2 | 1 | 2 | 0 | 2 |
| **4 – Severe disability** | 0 | 0 | 0 | 0 | 0 | 2 | 0 | 3 | 0 | 2 |
| **5 – Coma or vegetative state** | 0 | 0 | 0 | 0 | 0 | 0 | 0 | 1 | 0 | 0 |
| **6 – Brain dead** | 0 | 4 | 0 | 0 | 0 | 0 | 0 | 0 | 0 | 0 |

**S5. Characteristics and functional outcome of participants and nonparticipants of the neuropsychological assessment**

| Overall  (n = 55)^a^ | Participants  (n = 26) | Non-participants  (n = 29) | p-value |
| --- | --- | --- | --- |
| **Characteristics** |  |  |  |
| Age at event | 2.7 [1.9, 5.6] | 2.4 [1.8, 5.4] | 0.613 |
| Male gender | 16 (61.5) | 18 (62.1) | 1.000 |
| Bystander BLS | 23 (88.5) | 24 (85.7) | 1.000 |
| CPR duration (minutes) | 5.0 [2.0, 15.0] | 3.5 [1.8, 13.3] | 0.409 |
| First lactate (mmol/L) after ROC | 4.8 [2.7, 13.2] | 6.5 [3.7, 14.3] | 0.222 |
| First pH after ROC | 7.2 [6.9, 7.2] | 7.2 [7.0, 7.3] | 0.774 |
| First temperature at ED | 32.4 [30.4, 36.0] | 34.2 [33.1, 36.3] | 0.486 |
| SES parents |  |  | 0.605 |
| - 1 (low) | 4 (15.4) | 6 (22.2) | 0.776 |
| - 2 (intermediary) | 17 (65.4) | 14 (51.9) | 0.471 |
| - 3 (high) | 5 (19.2) | 7 (25.9) | 0.800 |
| **Functional outcome** |  |  |  |
| Age at longest general follow-up (years)* | 10.2 [5.3, 12.7] | 4.2 [2.1, 8.1] | **0.002** |
| PCPC at longest follow-up | 2.0 [1.0, 2.0] | 1.0 [1.0, 3.5] | 0.977 |
| FSS at longest follow-up | 6.0 [6.0, 7.0] | 6.0 [6.0, 10.0] | 0.065 |

Continuous variables are reported as median with interquartile ranges (IQR). Categorical variables are presented as number of subjects (n) and percentages (%).

^a^ 59 patients survived to hospital discharge; 4 patients died after hospital discharge due to complications due to event & of those 4 patients 2 died before any follow-up was possible.

BLS = basic life support, CPR = cardiopulmonary resuscitation, ROSC = return of spontaneous circulation, SES = socioeconomic status, PCPC = pediatric cerebral performance category, FSS = Functional Status Scale.

**S6. Patient and event characteristics: unfavourable outcome split**

|  | Survival with unfavourable outcome (PCPC 4-5) n = 8 | No survival  (i.e. PCPC 6) n = 44 | Missing |
| --- | --- | --- | --- |
| Patient characteristics | | | |
| Age at event (years) | 2.1 [1.6, 3.0] | 3.8 [2.3, 5.9] | 0 (0.0) |
| Male gender | 7 (88%) | 31 (70%) | 0 (0.0) |
| Pre-existing illness | 1 (13%) | 7 (16%) | 1 (2.0) |
| SES parents |  |  | 3 (6.0) |
| *- 1 (low)* | 0 (0%) | 16 (36%) |  |
| *- 2 (intermediary)* | 7 (88%) | 21 (48%) |  |
| *- 3 (high)* | 1 (13%) | 4 (1%) |  |
| Event characteristics | | | |
| Events witnessed | 0 (0%) | 4 (1%) | 1 (2.0) |
| Bystander BLS | 6 (75%) | 26 (59%) | 2 (4.0) |
| CPR duration (minutes) | 75.0 [12.0, 85.0] | 60.0 [30.0, 94.0] | 14 (14.1) |
| ECPR |  |  | 0 (0.0) |
| ROC | 8 (100%) | 26 (59%) | 0 (0.0) |
| *- At scene* | 3 (38%) | 12 (46%) |  |
| *- At ED* | 5 (63%) | 14 (54%) |  |
| First pH after ROC* | 6.6 [6.6, 6.9] | 6.8 [6.5, 6.9] | 2 (4.0) |
| First lactate _(mmol/L)_ after ROC* | 16.5 [14.1, 21.3] | 15.0 [14.6, 18.3] | 2 (4.0) |
| First temperature at ED | 30.6 [28.8, 32.7] | 30.1 [28.4, 32.8] | 4 (8.0) |
| Post-ROC ECMO | 1 (13%) | 6 (14%) | 0 (0.0) |
| Temperature management** | 8 (100%) | 18 (41%) | 0 (0.0) |
| Best GCS in the first 24 hours after ROC |  |  | 0 (0.0) |
| *- Eyes* | 1.0 [1.0, 4.0] | 1.0 [1.0, 1.0] |  |
| *- Motoric* | 2.0 [1.0, 5.0] | 1.0 [1.0, 1.0] |  |
| *- Verbal (intubated)* | 8 (100%) | 42 (95%) |  |
| Pupillary reflex present |  |  |  |
| *- At admission* | 5 (63%) | 9 (20%) | 2 (4.0) |
| *- First 24 hours* | 8 (100%) | 13 (30%) | 6 (12.0) |
| *- At discharge/ WLST* | 8 (100%) | 7 (16%) | 8 (15.0) |
| Follow-up (FU) | | | |
| Survival to hospital discharge | 8 (100%) | 4 (1%) | 0 (0.0) |
| FU interval (years) | 3.9 [1.2, 8.1] | 1.2 [0.7, 5.2] | 0 (0.0) |
| Age at longest FU | 6.0 [2.9, 11.5] | 4.8 [3.2, 8.1] | 0 (0.0) |
| PCPC at hospital discharge*** | 4.0 [4.0, 5.0] | 5.0 [4.0, 5.0] | 0 (0.0) |
| PCPC at longest FU*** | 4.0 [4.0, 4.0] | N.A. | 0 (0.0) |
